# Supporting quantitative skills in biomedical science with smart worksheets: intentions, impact and barriers to engagement

**DOI:** 10.3389/bjbs.2026.15078

**Published:** 2026-06-29

**Authors:** Leanne Williams, Emily Coyte, Sue Jones, Stephany Veuger

**Affiliations:** 1 School of Life Sciences, University of Warwick, Coventry, United Kingdom; 2 LearnSci, Bristol, United Kingdom; 3 Education, Institute of Biomedical Science, London, United Kingdom; 4 School of Geography and Natural Sciences, Northumbria University, Newcastle upon Tyne, United Kingdom

**Keywords:** action and barriers, asynchronous personalised learning, biomedical sciences, confidence and student outcomes, diverse students

## Abstract

**Introduction:**

The growing diversity of university student populations highlights the critical need for inclusive support strategies. Student outcomes often remain linked to socio-economic factors and Level 3 qualifications, challenging universities to create equitable learning environments, particularly for foundational skills like quantitative analysis. This study investigated the impact of widening participation (WP) characteristics on student engagement with, and perceptions of, novel asynchronous digital Smart Worksheets developed by LearnSci in collaboration with the Institute of Biomedical Science.

**Methods:**

Students (N = 779) enrolled on Biomedical Science modules in Level 3 to 6 at Northumbria University UK, were surveyed to gauge perceived calculation confidence, extracurricular commitments and intent to use the Smart Worksheets. Following deployment of the Smart Worksheets, students were surveyed again to correlate intention versus actual usage, explore the influence of WP characteristics and elucidate other factors that affected engagement.

**Results:**

Surprisingly, perceived course engagement and initial calculation confidence was generally high. Students with multiple WP characteristics, however, reported significantly lower calculation confidence (*p* = 0.002). While 93.9% of students expressed strong intent to use the resources, only 20% engaged. Further exploration revealed the primary barriers were resource awareness and time availability, with extracurricular commitments increasing from Level 3 to 6, culminating in 86.1% of Level 6 students having work or caring responsibilities. Regardless of initial confidence, students consistently rated the Smart Worksheet content as appropriate and their content as highly valuable.

**Discussion:**

These findings suggest that students’ extracurricular commitments are escalating throughout their university careers, challenging traditional assumptions about final year students being more autonomous and needing less support. Flexible, high-value resources are crucial for time-poor students, addressing engagement barriers that are primarily time and capacity-related. This study underscores the increasing need for comprehensive and clearly communicated support mechanisms for all students navigating an increasingly complex academic, financial and personal landscape.

## Introduction

### Learner diversity

Since 2000, the number of students entering higher education (HE) in the UK has steadily increased, reaching an annual total of more than 2 million undergraduate students from 2020 onwards. Between 2019/20 and 2023/24, undergraduate student numbers increased by 35% [1]. Universities and Colleges Admissions Service (UCAS) is the UK’s centralised organisation that manages applications to undergraduate degree courses. It reported at the end of 2024 that the proportion of UK 18-year-olds who would enter HE was 36% (an increase of 0.7% from 2023 and the first year-on-year increase since 2021) [2]. In the same time period, the number of academic staff in the UK (both teaching and research, full time and part time roles) only increased by 8% from 98,085 in 2019/20 to 106,285 in 2023/24 [3]. Recently, the ongoing rise in student numbers has coincided with many universities in the UK facing financial difficulties and reducing their number of academic staff [4]. Therefore, a balance must be struck between staff resources and successfully supporting diverse learners to achieve well.

Concomitantly, in the past 5 years, the number of students in the UK completing Level 3 qualifications other than traditional A-levels has also increased. In England, this rose from 27% in 2019/20%–35% in 2023/24 with a corresponding drop in A-levels from 73% to 65% [5]. The increase in non-A-level students entering HE is significant, particularly given the additional challenges faced by students who complete other Level 3 qualifications. Although the A-level alternative, Business and Technology Education Council (BTEC) Level 3 qualifications, are considered equivalent to A-levels for entry to UK BSc programmes, students who have BTEC only qualifications have double the chance of dropping-out of their studies, compared with those from an A-level only background (11% versus 6%) [6]. Further, it has been widely reported that in the UK, Level 3 qualification type and the grade or outcome achieved correlates with the qualification holder’s gender, age, ethnicity and indices of multiple deprivation (IMD) [7]. English indices of deprivation 2025 (IoD25), which includes the Index of multiple deprivation (IMD) enable local areas across England to be ranked from 1 (most) to 33755 (least) deprived. IMD rankings combine information from seven domains: income, education, employment, health and disability, crime, barriers to housing and services and living environment. Ranks are grouped into ten equal sized deciles with decile 1 being of the 10% most deprived and decile 10 being the least [8]. Higher Education Statistics Agency (HESA) data show that the proportion of undergraduate students (first time degree) who were awarded a first or an upper-second degree directly correlates with the HESA UK-wide area-based measures of deprivation. Degree achievement rises with a linear correlation from IMD decile 1 to decile 10, with a 16-percentage point gap in degree achievement between those in the most and least deprived deciles [9]. This study reports on work completed at Northumbria University, which has been committed to widening participation for many years. Northumbria University reports that the institution overall has a slightly lower proportion of students in IMD Q1 and Q2 and slightly fewer entrants who were eligible for free school meals, however, more than 62% of their UK undergraduate students are from areas of low representation in HE [10]. Whilst IMD provides a holistic view of deprivation, Tracking Underrepresentation by Area (TUNDRA) data is a more specific metric developed by the Office for Students (OfS). It focusses on HE participation, tracking school children into HE. It is ranked similarly to IMD with quintile 1 being lowest rate of entrance into HE and 5 being the highest [11]. Northumbria University has 36.8% students from TUNDRA quintiles 1 and 2, compared with the English provider average of 27.8%. It is commonly accepted that the broader challenges faced by students from widening participation (WP) backgrounds, who are encouraged to study at university, can impact negatively on their social mobility and ability to achieve well academically. Students who are care experienced, have caring responsibilities and those who must undertake paid employment alongside studying, all face complex challenges as they transition to HE and adjust to the requirements of university. The key transitional barriers faced by students from WP backgrounds can be summarised as follows: *Preparation:* lack of academic and cultural readiness for university life, which impacts on student confidence, engagement and sense of belonging [12]; *Participation:* reduced access to support and peer networks because of commuting or financial pressures that necessitate paid work, impacts on study time and networking opportunities; *Persistence:* Coping with additional pressures (financial, mental health, time, external responsibilities) that challenge concentration, attendance and retention [13]. It is therefore essential that academic staff have a good understanding of the prior learning experiences and external commitments of their students to be able to provide intentional support for them. Providing such individual and meaningful learning experiences must be carefully balanced against increasing academic staff workloads.

### Student belonging and engagement

Traditional Learner Centred Pedagogy (LCP) tools for effective student engagement commonly include synchronous activities such as scheduled sessions with real time engagement from students with peers and academic staff [14]. Such face to face (on campus and/or online) sessions can be used to foster a sense of belonging and community. Dynamic interactions and clarification in synchronous sessions can also positively impact a student’s motivation and accountability [15]. In-person and synchronous activities foster constructivist learning through active engagement and discussion [16] and the Socratic method through live discussions and collaborative learning which helps to develop critical thinking skills [17].

There is an abundance of literature demonstrating the correlation with the benefits described above and academic achievement [18]. However, despite these known advantages, the reduction in student ‘engagement’ particularly since the COVID-19 pandemic, has been a subject of much debate in higher education. The UK Engagement surveys (UKES) provide useful insights into student responses to key questions around engagement, skills development and time spent studying or completing other tasks. UKES data reveal interesting trends in student activities and engagement post-pandemic [19]. Across four of the seven indicators of engagement used, students reported engagement levels equal to or exceeding pre-pandemic levels. Students also reported interacting with academic staff more often during 2022 compared with any other year since 2018, although a significant number of students surveyed also expressed feelings of loneliness and a lack of connection with both peers and staff.

The proportion of students managing caring responsibilities alongside their studies was 34% and those undertaking paid work in addition to studying was 55% [19]. These UKES data align well with a comprehensive student engagement survey carried out in 2023 [20] by the independent think tank Higher Education Policy Institute (HEPI) and Advance HE, an HE member led agency. A significant factor was financial strain, with 76% of students reporting that the cost-of-living crisis affected their studies, including 26% stating their studies were impacted “a lot.” These issues disproportionately affect students from WP backgrounds, as undertaking paid employment reduces available time to engage with taught content, peers and academic staff [21].

Without addressing these disparities, our current teaching, learning and assessment strategies and student support mechanisms will fail to meet the needs of students from WP backgrounds. Collins-Warfield et al. outline the responsibility of universities to meet the specific educational needs of students from marginalised backgrounds, historically excluded or disadvantaged groups, those from low-income backgrounds and students who are ethnically minoritised [22]. They advocate for personalised learning, and more empathy and care for students to facilitate inclusivity in taught sessions. Developing personalised initiatives to foster self-confidence and autonomy for students alongside increasing workloads for both students and academic staff is, however, proving to be an increasingly difficult balance. Self-regulated learning (SRL) as established by Zimmerman is an active, dynamic process that involves the intersection of motivation, method, time, physical environment, performance and self-reflection [23, 24]. If we fail to provide opportunities for students to explore these domains both independently and with our guidance, particularly in this landscape of high assessment burden and time poor students, we risk engagement becoming ever more intangible.

It is vital that the HE sector can distinguish between low student attendance and engagement, identify their respective causes, and critically examine any causal links to academic achievement. As outlined above, it is evident that learning culture within UK HE institutions is shifting, meaning students may no longer engage in ways traditionally anticipated by, or embedded within, our established educational frameworks. Consequently, providing meaningful and motivational supporting resources, increasing optionality, and individualising learning pathways where feasible, is essential to enhance SRL opportunities. As explored in this study, asynchronous digital tools and strategic choice offer a promising solution for building students’ confidence, and competence, particularly prior to high stakes assessments.

### Regional and institutional context

This study was conducted at Northumbria University (NU), which has a strong and sustained commitment to social mobility, with almost 40% of its current students recruited from low participation backgrounds. Broadly, the student body at NU reflects UK data reported by HESA, with students being recruited from increasingly diverse backgrounds over time. High numbers of undergraduate full time students from the North East (NE) of England choose to study at a local university. HESA data show that 54% students from Northumberland, 66% from Redcar/Cleveland, 68% from Middlesbrough and 74% from Hartlepool choose to study in the NE [25]. Given this context, it is useful to consider factors such as health, education and the economy in the NE region of England that are mirrored in the student population. The percentage of the population in the NE with GCSEs in English and Maths is 1.9% lower compared with the rest of England, and the percentage with a Level 3 qualification is 4.6% lower compared with Great Britain. The NE region also has serious health inequalities, with female healthy life expectancy 4.4 years lower and male healthy life expectancy is 4.6 years lower compared with the rest of England [26].

In 2023/24 NU had 20,580 full-time UG students, with 54% female and 46% male students [1]. The majority (over 80%) of UG students at NU are under 21 years old, 20% are international students and in terms of ethnicity, nearly 80% of students identified as white, approximately 5% as Asian, 2% as Black, and 2% mixed ethnic background, which broadly represent the demographics of the NE region. Additionally, 10% of students at NU were eligible for free school meals and 20% home students are from the most deprived IMD quintiles 1 (Q1) and 2 (Q2). Finally, 15% of students at NU identify as having a disability which aligns with the national data of 15%–18% students with a declared disability between 20202/1 to 2023/24 [1]. Importantly, the number of students studying at NU who completed Level 3 qualifications other than A-levels, is higher than the national data (35%), at almost 50% [27].

During enrolment in 2024/25, approximately 70% of eligible students completed the NU diversity monitoring form, 88.6% respondents self-identified as belonging to one or more widening participation groups. These groups include (but are not limited to) students who identify as Black, Asian or Minority Ethnic, Care Leaver, Commuter Students, Disabled, First-Generation, Eligible for Free School Meals, Low Participation Neighbourhoods (LPN), or Young Carer [10]. The NU Access and Participation Plan (2024–2028) recognises issues in attainment for the most disadvantaged student groups: “For continuation, there has been a sharp decline, with the gap between TUNDRA Q1 and Q5 growing from 3.3% points (2019/20 entrants) to 5.9% points (2020/21 entrants), the gap between IMD Q1 and Q5 growing from 2.4% points (2019/20 entrants) to 8.0% points (2020/21 entrants) and the gaps between those not eligible for free school meals and those who are eligible growing from 1.8% points (2019/20 entrants) to 5.3% points (2020/21 entrants). In all cases, these growing gaps can be attributed to the rate of decline in the performance of the more disadvantaged group” [10].

In 2023/24 the Department of Applied Sciences at NU was flagged for both retention (a proxy for continuation) and degree award (number of Level 6 students achieving 2:1) for differences between IMD Q1/2 and Q3/4/5 students. These data provided the incentive to find and implement interventions to better support the diverse student cohorts. The largest programme in the Department, BSc Biomedical Science, has many quantitative components in the curriculum, as required by QAA benchmark statement [28] and IBMS accreditation criteria [29]. Academic colleagues commonly report that students often struggle, not only because of skill gaps, but often a deep rooted “fear” of maths [30] that needs to be overcome. This phenomenon is particularly noticeable at Northumbria University, where the Biomedical Science programme team consistently highlight that students on their modules struggle to complete data processing and problem-solving activities.

Strategies to support the diverse student cohort at NU is a strategic priority, with an institutional objective to “enhance student outcomes and close outcome gaps between student demographic groups”. This study focuses on intentional interventions to support students in all levels of study who are completing modules from the BSc (Hons) Biomedical Science programme at Northumbria University, accredited by the Institute of Biomedical Science (IBMS). Students can enrol on a foundation year at Level 3 which feeds into the programme at Level 4 and there is an optional placement year between year 2 (Level 5) and 3 (Level 6). All students study 120 credits per year, and each module has a tariff of 20 credits, except for the final year Level 6 research project which is a year-long 40 credit module. Admission to the course requires grade 4 in mathematics at GCSE. In the academic year 2024/25, 387 students were enrolled on the BSc Biomedical Science programme with 66% female students across the whole programme, similar to national data for Biomedical Sciences programmes at 70% female students [31]. The focus of the work presented here is the implementation of tailored and flexible digital tools to support student learning in all levels of undergraduate study. In parallel with improving confidence and competence, the hope is to increase engagement with core learning material and improve student achievement. LearnSci is an Edtech company that specialises in the development of digital resources to support technical and practical skill acquisition. Working in collaboration, the IBMS and LearnSci developed a series of quantitative skills Smart Worksheets (SWs) framed within a biomedical science context and aligned to the IBMS accreditation criteria and UK Quality Assurance Agency (QAA) Subject Benchmark Statement 2023 [28]. The objective is to bridge the gaps in prior knowledge and support students with core skills.

### The intervention

The LearnSci Quantitative Skills (QS) for Biomedical Sciences Smart worksheets (SW) are a series of five resources designed to support students with the numerical components of their course. They were developed in collaboration with the IBMS to ensure their quality and relevance to students studying Biomedical Science programmes.

The five topic areas are as follows:

- QS1 - Fundamental Numeracy
- QS2 - Units and Unit Conversions
- QS3 - Scientific Formula
- QS4 - Data Visualisation and Graphs
- QS5 - Averages, Spread and Precision

**FIGURE 1:**
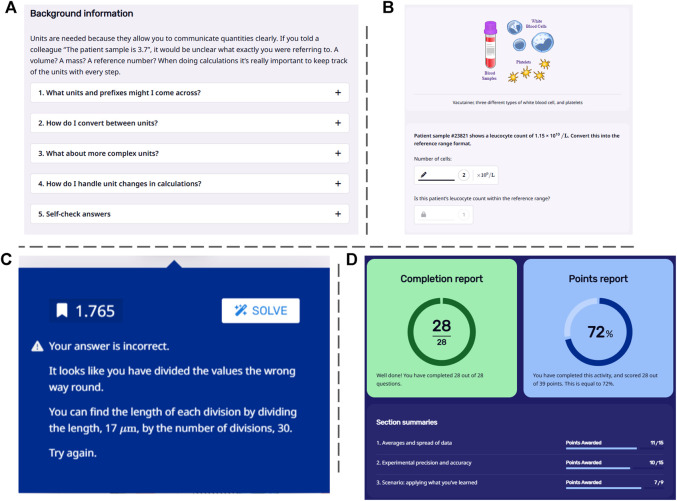
Selected example aspects of the Quantitative Skills for Biomedical Scientists Smart Worksheets, including: **(A)** background information, **(B)** an example question, **(C)** example feedback, and **(D)** reporting.

The QSSWs have a variety of features designed in accordance with a robust pedagogical framework founded on constructive alignment [32] and hierarchical cognitive learning outcomes (LO) [33–35] that offers opportunity for individual learning pathways (Figure 1). Each SW includes preparatory background information and self-check questions for preparation (Figure 1A) and more in-depth scenarios to reinforce the quantitative topic areas (Figure 1B). The questions have relevant contexts, but completion does not rely on recalling specific topic knowledge beyond what is provided in the questions. This design was intentional, to increase the relevance of the resource content to the students while allowing it to be usable by students from the start of their degree programme.

Each question provides instant feedback on incorrect answers, which cater for specific errors and common misconceptions (Figure 1C). This is a vital component of the SW technology as it encourages exploratory constructive learning. All SWs include images, tables, graphs, authentic data and interactive components to motivate and engage students. An end report shows the student’s completion and score; overall, by section and by learning outcome (Figure 1D). Finally, self-reflection is encouraged, and feedback can be reviewed via a ‘Timeline mode’, allowing the students to review and repeat questions and answers.

Smart Worksheets can be embedded within the University’s chosen Learning Management System (LMS) and can be applied to a variety of learning, assessment and subject contexts. The SW adaptive technology can be used formatively to offer flexible and efficient feedforward strategies, e.g., workshop design [36, 37] and low stakes learning experience for students to provide unique user experience that improve grades [38, 39] and close awarding gaps [40].

### Aims and objectives

This study primarily aims to enhance students’ quantitative skills development, by addressing both the cognitive and affective domains of learning [41]. While cognitive measures track technical proficiency, the affective domain, specifically perceived confidence, was selected as a critical metric because it functions as a gateway to skill acquisition. Grounded in “Self-efficacy theory” [42, 43], this approach recognises that a student’s belief in their numerical capability can influence their persistence and engagement with complex biomedical data. By evaluating initial perceptions and confidence levels, the research identifies psychological barriers such as “maths anxiety” that can impede cognitive progress in STEM [44]. Furthermore, the study investigates how key widening participation (WP) factors, including first-in-family status, pre-university education, gender, international student status, and external employment or caregiving responsibilities impacts on student engagement experiences.

To further this aim, the study examines the implementation and impact of five new Quantitative Skills for Biomedical Science Students Smart Worksheets (SWs), developed in collaboration between LearnSci and the IBMS. These online, interactive resources address topics fundamental to analytical biomedical science, designed to cultivate essential calculation and data handling skills within relevant and authentic scenarios. Each SW provides structured guidance, unique data components for personalised learning and practice, and offers instant, targeted feedback to improve learning efficiency and support feed-forward strategies. The SWs were provided as non-compulsory, supplementary resources to students across several modules at all levels (Level 3 to Level 6) delivered on the BSc Biomedical Science programme at Northumbria University.

The secondary aim of this study is to elucidate the effectiveness of these LearnSci SWs as an optional study support mechanism and to identify optimal deployment strategies for supporting diverse student cohorts.

## Materials and methods

### Overview

This study primarily involves quantitative analysis, with some qualitative exploration on the subjective experience of students in Levels 3 to 6 enrolled on BSc Biomedical Science programme modules. Ethical approval to conduct the study was obtained from Northumbria University’s ethics committee [7860] prior to commencing the work.

Students were provided with access to the five SWs through the LMS, Blackboard, during semester one in the academic year 2024/25. All SWs were offered as non-compulsory supporting resources to students on several modules (Table 1) of the NU BSc Biomedical Science programme. The modules were chosen for their relevance to quantitative skills and include Level 3 scientific and numeracy skills; Level 4 core practical and analytical skills, a Level 5 research and analysis and Level 6 capstone research projects.

**TABLE 1:** Semester one 20 credit modules running September to December 2024. The total number of students per module and key demographic data. The project module (Level 6) is a 40 credit year-long module and the level 4* module is a module taken by a largely (but not exclusively) international cohort in advance of transferring to the medical degree at NU.

Level	Module	Total number	Overseas	Home	Male	Female
3	Scientific and numeracy skills	159	46	113	66	93
4	Practical skills	253	31	222	88	165
4*	Practical skills for health sciences	73	71	2	33	40
5	Bioscience research and analysis	173	26	147	66	107
6	Biomedical science research project	121	8	113	38	83

Student recruitment for this study was facilitated by a face-to-face session in each module listed in Table 1 during week one of semester one in September 2024, to introduce the SW resources and the aim of the study. A follow-up email from the e-learning portal for each module listed in Table 1 was also sent but there was no solicitation of participants. Student surveys were completed before (Survey A) and after (Survey B) exposure to the Quantitative Skills SWs, and further analysis of student usage and perceptions of these resources was conducted. It was made clear that participation in this study was voluntary, participant information was provided, and a consent question was asked at the start of each survey.

Students were advised that they were not being assessed on their performance in the SWs and that they had unlimited attempts. A second face-face session was held in each module during week 11 of Semester one in December 2024 to survey the students on their experiences of using the SW resources. All question responses were anonymous and (after the consent question) optional to encourage students to participate at whatever level they felt comfortable. As a result, the sample size between questions varied.

### Survey A

The first survey (Supplementary Material 1) was hosted on SurveyMonkey and open to students at the start of the academic year from 30th September - 23rd October 2024 via a QR code and link. It had 11 questions and was designed to capture a snapshot of students’ perceived confidence in quantitative skills, their level of course engagement, their intention to use optional supporting resources, plus the factors that would encourage or discourage engagement. Demographic questions and extracurricular commitments were also explored to enable analysis of widening participation cohorts. A total of 387 students took Survey A across the targeted modules in Levels 3 to 6.

### Survey B

The second survey (Supplementary Material 2) had 22 possible questions, and the overall structure was similar to Survey A, although the content was different. After the consent question, students were asked if they had used all, some or none of the Quantitative Skills SW resources. Subsequent questions were revealed depending on the student’s answer, for example, those who had completed some or all were asked for their perceptions of the resources, and those who had not completed any resources were asked to provide reasons why. At the end of Survey B, all students were asked for the same demographic information as in Survey A.

Survey B was open to students from 25th November 2024–19th February 2025. Up to 155 student responses were obtained across Level 3 to 6. All question responses were anonymous and (after the consent question) optional to encourage students to participate at whatever level they felt comfortable. As a result, the sample size between questions varied. As the semester progressed, in person attendance and engagement with all modules decreased, limiting the impact of timely reminders and therefore the number of respondents for survey B is lower than that of survey A.

### Data analysis

Quantitative survey data were analysed with SPSS version 30. Due to their broad but short and shallow nature, open comment texts on encouraging and discouraging factors were explored using content analysis [45] with a focus on identifying surface level components and counting occurrences of frequent and recurring words, themes or concepts.

## Results

### Student demographics

A total of 779 students from the foundation year and across Level 4 to Level 6 were enrolled on the modules used in this study. The response rate for participation in Survey A was an impressive 42%, with the highest level of engagement observed for Level 4 students (150 participants) decreasing in Level 6 (72 participants); Level 5 (56 participants) and Level 3 (49 participants) respectively. The response rate for survey B was much lower at 20%.

**FIGURE 2:**
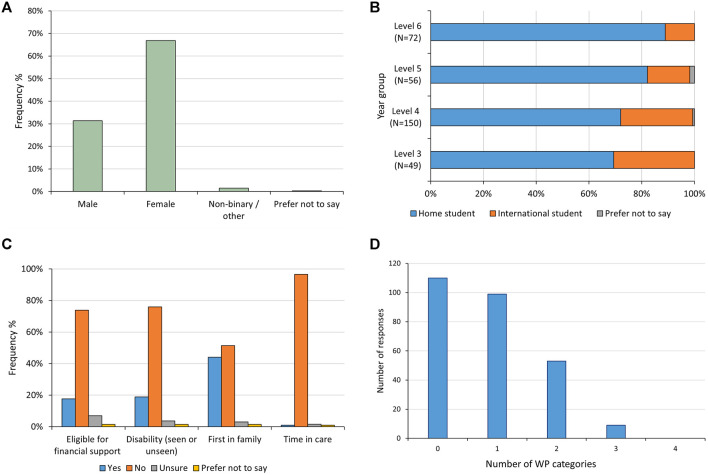
Participant demographics in Survey A. **(A)** Participant responses to a question asking for their gender (N = 329) demonstrates a higher proportion of female-identifying students, in agreement with the whole cohort data. **(B)** The proportion of home versus international students across the programme highlights a clear trend: a progressive decrease in the proportion of international students as the academic years progress. **(C)** Frequency with which participants selected various widening participation (WP) categories (N = 271). Whilst students identifying with WP characteristics are less frequent overall, there is a sizable number of students who do identify with these characteristics, with the experience of being first in family to attend university being particularly prominent. **(D)** The intersectionality of WP categories amongst participants indicates that most students align with a single characteristic, a notable subset recognises themselves within two or even three WP categories.

Across Survey A participants, the overall gender split was 66.9% female, 33.3% male, 1.5% non-binary and 0.3% preferred not to say (Figure 2A) which is representative of the cohort overall. The number of international students in Level 3 is higher than the sector average at 30.6%, but this reduces across the subsequent levels of study (27.3% in level 4, 16.1% in Level 5% and 11.1% in Level 6; Figure 2B). The international student data can be explained by students transferring internally to the medicine degree at Northumbria University after Level 4 of the BSc Biomedical Science programme. To investigate the intersectionality of external influencing factors on students’ learning capital, widening participation (WP) characteristics were explored. A list of the four core WP categories (socio-economic background, disability, first in family status and care experience) was included in the survey for students to select all those they identified with (Figure 2C). Of those who completed the questions, 161 (59.4%) of students considered themselves in one or more WP categories (Figure 2D). Whilst Survey B had a lower sample size, participants had similar demographic characteristics, ensuring the data was not over or underrepresenting any cohorts (data not shown).

### Engagement, confidence and intention

**FIGURE 3:**
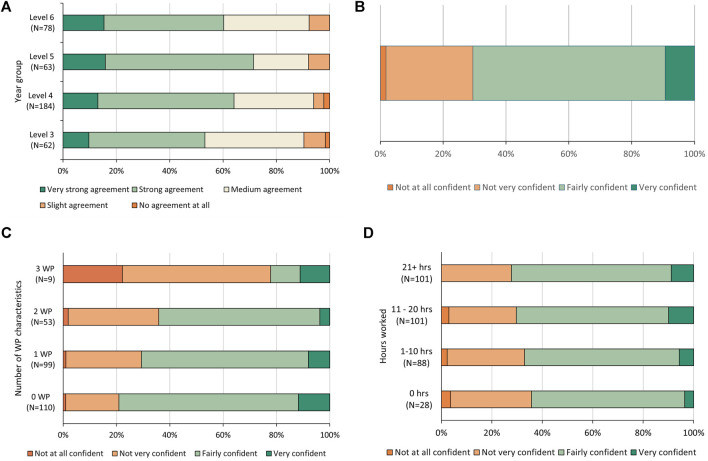
Course engagement, perceived confidence and intersectionality: **(A)** I feel engaged with the content of my course (N = 387), responses demonstrate a similar trend across all levels. There was no significant difference (Kruskal-Wallis Test, H = 6.545, p = 0.091). **(B)** How confident do you feel when answering calculation questions in your course? (N = 387). **(C)** Perceived confidence and number of selected WP categories (N = 271). The higher the number of WP characteristics, the lower the confidence: Kruskal-Wallis H = 14.507, p = 0.002. **(D)** Perceived confidence in answering calculation questions and number of hours worked (N = 318; top highest hours to bottom lowest hours).

Student disengagement is a widely acknowledged and increasingly reported problem across the sector [46], with several contributing factors cited as potential causes [18]. To assess student perceptions of engagement, we asked students how much they agreed with the statement: “I feel engaged with the content of my course”. Contrary to prevailing concerns, the self-reported levels of engagement were high with Level 5 students demonstrating the most optimism (Figure 3A).

Again, rather surprisingly, students generally considered themselves to be fairly confident with calculation questions on their course (Figure 3B). There was no significant difference in reported confidence Kruskal-Wallis Test (H = 6.545, *p* = 0.091, asymptotic) between levels of study (not shown). This higher-than-expected general confidence may be related to participant interpretation of the term ‘calculation questions’ which may be perceived as performing short calculations, rather than mathematics and analysis in general as we intended. International students (N = 73) rated themselves as having slightly higher confidence levels than home students (N = 252) but there was no significant difference: U = 8,099.5, *p* = 0.071 (exact 2-tailed). Male students (N = 103) reported feeling significantly more confident than female students (N = 220): U = 9,910.5, *p* = 0.036 (exact 2-tailed). When confidence rating was correlated with the number of self-selected WP characteristics, however, there was a highly significant difference in perceived confidence. The more WP characteristics, the lower the confidence level reported, Kruskal-Wallis H = 14.507, *p* = 0.002 (exact; Figure 3C). Interestingly, there was a small but steady trend of students acknowledging a slight increase in confidence relative to their number of hours in paid work each week, but this was not significant (Figure 3D).

**FIGURE 4:**
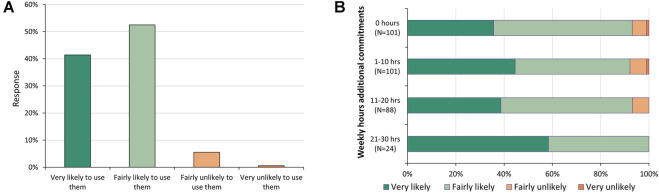
Perceived engagement intention: **(A)** If an optional set of resources aimed at developing your calculation skills in biomedical science contexts was available to you, do you think you would use them? (N = 343) 93.5% responses were very or fairly likely to use the resources. **(B)** Responses split by hours of additional commitments: Those with the most hours of commitments indicate a higher tendency for very and fairly likely to use the resource, however, there was no significant difference (Kruskal-Wallis H = 6.704, p = 0.081 (exact); Top lowest hours to bottom highest hours).

When offered bespoke resources focussed on consolidating calculation skills directly relevant to high stakes assessment, students express very high levels of intention to use such resources with 93.9% saying they were ‘very likely’ or ‘likely’ to use them (Figure 4A). The general pattern is comparable across year groups with Level 5 and 6 students showing slightly more intention to use such resources, but there was no statistically significant relationship (Kruskal-Wallis Test, H = 7.291, p = 0.063 (exact)). Students with lower self-reported confidence in answering calculation questions did, however, state that they were more likely to engage with optional resources designed to support them. This is statistically significant, Kendall’s tau = 0.-112, p = 0.028 (two-tailed) and suggests that students who stand to gain the most are aware of this and demonstrate a significant intention to engage. The relationship between intention to use the resources and extracurricular commitment, showed an increased likelihood with those working/caring the most hours, but this was not statistically significant (Kruskal-Wallis H = 6.704, p = 0.081 (exact) (Figure 4B).

To explore the conditions to support maximum engagement, we asked students what factors would encourage and discourage them from using the SWs provided. The categories and frequency of suggestions following a content analysis is shown below (Table 2). Encouraging factors focused on ease of access and use; relevance to course content and skills; learning efficiency such as staged progression, repetition and quality of feedback. Conversely, difficulty in finding the resources, navigating and using the resource; irrelevance and complexity of content were discouraging factors. Interestingly, for some students, compulsory use was seen as a barrier.

**TABLE 2:** The ten most prevalent student-reported factors that encourage and discourage the use of optional supportive resources to support calculation skills derived by content analysis of all student responses (N = 168 for encouraging, N = 150 for discouraging).

Factors encouraging usage		Factors discouraging usage	
Category	# of responses	Category	# of responses
Easy to find/access	34	Difficult to use/navigate	23
Easy/straightforward to use	18	Too long/too much content	17
Relevant	16	Too difficult or complicated	17
Practice opportunities	15	Not relevant	13
Feedback provided	15	Difficult to access/find	13
Interactive components	14	Lacking explanation support	11
High quality explanations	12	Cost present	7
Skill developments	11	Boring	6
Staged/stepwise components	11	Compulsory/for credit	6
Available on module site in VLE	9	Nothing	6

### Action and barriers

Following the in-person introduction to the Quant Skills SWs and them being made available, usage data was recorded. In total 25.3% (N = 197) of 779 students engaged with at least one of the five resources (started or completed). 5.6% used all five SWs, with 10.2% of students who completed one SW re-engaging with the same resource at least once more. When usage data were split into levels of study, highest SW engagement was from Level 5, with 45.1% using one or more SW, then Level 3 at 31.4%; Level 4 at 20.9% and only 0.8% engagement by Level 6 students.

**FIGURE 5:**
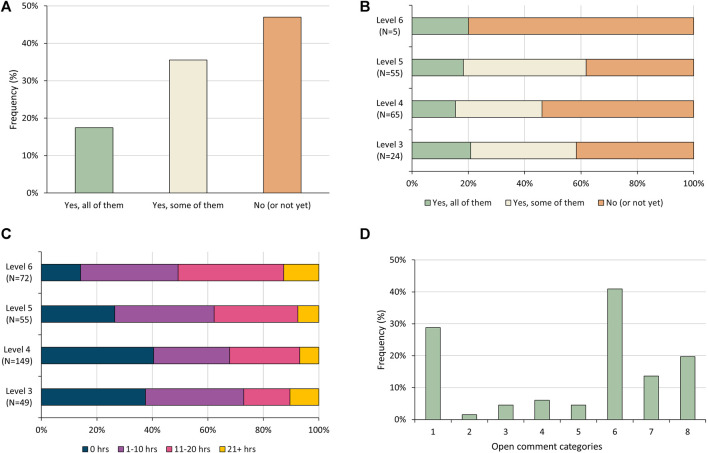
Smart Worksheet usage and barriers to engagement. **(A)** Have you used the quantitative skills Smart Worksheets? (N = 149). **(B)** By Level of study: Level 3 and 5 appear to have engaged more, however, the data are not significant (Level 6 excluded N = 5). **(C)** On average, how long do additional commitments (including paid work and/or caring responsibilities) currently take per week during term time? Extracurricular commitments significantly increase with year group: Kendall’s tau = 0.170, p < 0.001). **(D)** Why did you not use the Quantitative skills Smart Worksheets? This open comment question was analysed with seven key findings emerging. 1) I did not have time, 2) They looked too difficult, 3) too easy, 4) They did not look interesting, 5) I could not open or use them, 6) I did not know about them, 7) I could not find them 8) Other (non-specific answers).

Survey B was used to correlate actual usage data with claimed use of the Quant Skills SWs. 17.5% of 149 students who answered this question had used all five SWs, 35.6% had used at least one and 47.0% (at the time of compiling the data) had not used any (Figure 5A). There were some differences between students in different levels here too (Figure 5B), with Level 4 being less engaged (46.2% reporting using all or some of the SWs compared with 58.4% of Level 3% and 61.8% of Level 5). The Survey B respondents may not be fully representative of the whole cohort in this regard, but those who responded still offered valuable insight. It is worth noting that only five Level 6 students took Survey B. Combined with the low SW usage, this indicates very low overall engagement from this year group.

Given that 93.9% of Survey A respondents specified they were ‘very likely’ or ‘likely’ to use the resources, it is apparent that there was a significant discrepancy between the students’ intention to use and subsequent action. These data offered a valuable opportunity to explore the complexity surrounding why students do not or cannot engage with resources that could improve relevant skills.

Participant responses from Survey A (N = 325) indicate that over 86.1%; 74.6%; 58.4% and 63.3% of Level 6, 5, 4 and 3 students respectively have weekly additional commitments. With hours significantly increasing as the years progress (Kendall’s tau = 0.170, p < 0.001). Level 6 students are working/caring more hours than any other year group, with 12.5% working/caring over 21 h a week (Figure 5C). The impact of external commitments could therefore account for increased time pressure and justify why time limitation was one of the main reasons given for not engaging (Figure 5D) “I did not have time” (28.8%) and “I did not know about them” (40.9%). The open comment text for those who selected “Other” (19.7%) was primarily composed of “I forgot.”

### Timing and value

**FIGURE 6:**
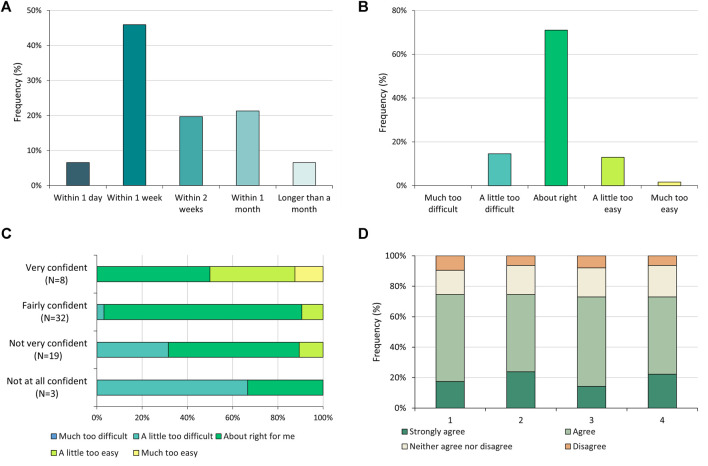
Smart Worksheet usage, pitch of content and impact. **(A)** How soon after hearing about the Quantitative skills resources did you start to use them? 93.44% of usage was within a 1-month period, with highest frequency of use being within the first week of being told at 45.9%. **(B)** What did you think of the overall challenge level of the Quantitative Smart Worksheets? 14.52% found the sheets a little too difficult; 70.97% about right; 12.90% a little too easy and 1.61% much too easy. **(C)** Challenge level split according to confidence in calculation ability **(D)** Regarding broader impact, how much do you agree that the quantitative skills resources 1) helped me identify my strengths and weaknesses (74.6% agree or strongly agree); 2) made me feel more confident in my calculation abilities (74.6% agree or strongly agree); 3) helped me engage more with the module overall (73.02% agree or strongly agree); 4) helped me feel more prepared for future assessments (73.01% agree or strongly agree).

A small percentage of students (6.6%) used the SWs within a day of being introduced to them, most students started using them within a week (45.9%), with substantial usage continuing within a 2 week and 1 month period. After that point, usage dropped substantially (Figure 6A). In terms of the pitch and level of content within the SW it was reassuring that the majority (71.0%) found the level of the SWs to be about right, and a symmetrical split between “a little too difficult” (14.5%) and “a little too easy” (12.9%). No respondents claimed the resources were “much too difficult” and only 1.6% considered them too easy (Figure 6B). As might be expected, the more confident students had purported to be with calculations (Figure 3B), the less difficult they claimed to find the SWs (Figure 6C). This is statistically significant according to Kendall’s tau correlation (0.442, p < 0.001, N = 62). However, as only one of these groups reached over 30 students, statistical reporting may not be reliable. Interestingly, 87.5% of those who were “fairly confident” and 50.0% who were “very confident” regarded the SWs as just about right (Figure 6C). The authors acknowledge that confidence does not always relate to ability, however, the value of the SWs was high for all abilities. This is supported by 73.0%–74.6% of students consistently agreeing or strongly agreeing that the SWs had broader impact on their learning: helping them identify strengths and weak points, feel more confident in calculations, engage more in the module overall, and feel more prepared for future assessments (Figure 6D). These data provide key indicators of how we might utilise relevant formative upskilling to help students feel more connected and engaged with their programme.

**FIGURE 7:**
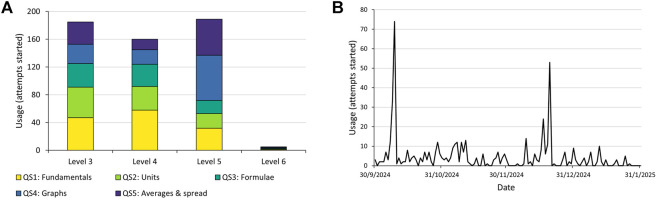
Choice and strategic use. **(A)** Individual Smart Worksheet usage data versus level of study. **(B)** Smart Worksheets attempted against time (30^th^ of September 2024 to 31st January 2025).

There are many factors that can underpin how and why students might engage with the SWs and these could have been explored further through focus groups. Unfortunately, the lack of student participation did not allow this to be pursued. Detailed analysis of each of the SWs demonstrates some interesting trends when split by level of study (Figure 7A). Level 3 and 4 students appear to work through the sheets systematically with usage dropping off as they progress. At Level 5, however, students appear to be more strategic in their selection of the SWs that they use. The highest usage was QS4 (Graphs) and QS5 (averages and spread). This may indicate that the first three sheets cover content students felt more comfortable with, or that they were influenced by the timeliness of a credit weighted assessment that required graphing and understanding y = mx + c for Level 5 (first spike; Figure 7B). After receiving results and feedback for a numeracy skills assessment and an in person reminder of both the resources and survey B, a spike in usage was seen for Level 3 students (second spike; Figure 7B).

**FIGURE 8:**
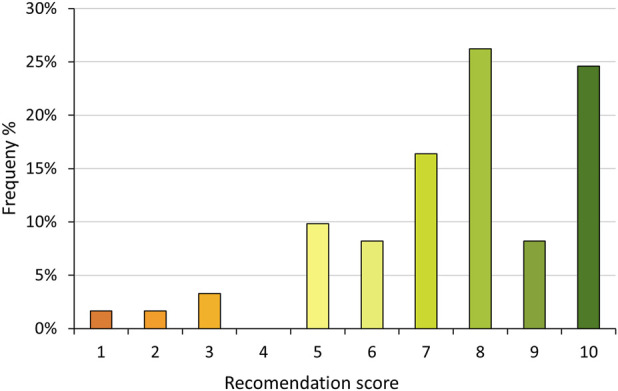
Quant Skills Smart Worksheet Recommendation Rating. On a scale of 1–10 with 1 being low and 10 being high, how much would you recommend the Quantitative Skills Smart Worksheet resources to other students on a similar course.?

Finally, students were asked to rate how much they would recommend the SWs (10 being high), 6.6% of students rated the sheets 1 to 3; 34.4% selected 5 to 7 and 59.0% 8 to10 (Figure 8). Overall, these ratings reflect the data from (Figures 6B,D) that the level of the SWs content is pitched effectively and had a positive impact on students’ learning.

## Discussion

Strategic approaches that consider the diverse needs and experiences of students are essential to create a more equitable and accessible higher education (HE) landscape for all learners. Beyond ensuring access, institutions must also address the increasing requirements of retention and success by providing inclusive teaching practices, targeted support, and flexible learning pathways. However, financial constraints and work burden can leave little room for implementing innovative personalised learning journeys for students. This study sought to understand the benefits of embedding constructively aligned [32] resources with course content and assessment components, to support transitional barriers and enhance the confidence and engagement of existing students undertaking Biomedical Science modules. The Quantitative Skills Smart Worksheets (SWs) were intentionally designed to familiarise and upskill students in key quantitative concepts, principles and theories. The series of five SWs used here cover fundamental concepts such as numeracy, units, formulae, data visualisation and graphs that collectively underpin data collection, presentation, analysis and interpretation skills.

LearnSci SW resources are valuable for improving skills, confidence and enhancing success [38–40]. They can be used to foster an inclusive and supportive learning environment, ensuring the programme evolves to meet student needs and enable every student to achieve their full potential. They also support the requirement to embed skills development at the heart of our programmes, as we seek to produce adaptable and resilient graduates, ready to make the most of future employment opportunities and emerging job markets. The availability of the SWs for students in Levels 3 to 6 studying modules delivered on the BSc Biomedical Science programme underpins and supports Northumbria University core principles and could support improvements in institutional key performance indicators (KPIs); 4 (graduate outcomes) 5 (student satisfaction) and 6 (continuation). Students in every level, with low to high calculation confidence found significant value in optional, flexible asynchronous learning resources. These skills-oriented worksheets can be introduced in Level 3 and 4 to build confidence and revisited in Levels 5 and 6 to re-familiarise students with key quantitative skills in the context of more complex data acquisition and evaluation.

### Key insights and take-home messages

This study reveals several critical insights regarding student engagement, support needs and the value of flexible learning resources in contemporary HE.

#### Student commitments are increasing, challenging traditional expectations

Students are juggling significant extracurricular work and/or caring commitments which are increasing as they progress through their degree programme. This challenges the historical/typical expectation that final year students should be ‘fully’ focussed on their studies and require less support.

#### Engagement is multifaceted and requires redefinition

While student perception of their course engagement is surprisingly high, the definition of engagement needs to be clarified to avoid over-simplification and align student and practitioner views. Students are negotiating barriers to engagement as traditionally expected (being in the room). Therefore, the message that engagement (i.e., attendance) correlates with achievement is less likely to impact students positively.

#### Flexible, high-value resources are crucial for time-poor students

Optional asynchronous resources like the SWs are highly valued, particularly by students with low calculation confidence and working long hours, as they offer flexibility and efficiency.

#### Barriers to engagement are primarily practical and communication-related

Despite high intention to use, actual engagement with optional resources can be hindered by lack of time, insufficient promotion and unclear guidance, as perceived by students.

#### Final year students are the most impacted

These students need support now more than ever as they navigate an increasingly complex and pressured landscape of study, work and/or have caring commitments. Students are fitting their degree around their lives rather than the other way around. Those students who can attend all timetabled sessions may now be only those in a privileged position.

### Learner diversity and evolving needs

As described in the Introduction, the student cohorts at Northumbria University (NU) are representative of Biomedical Sciences cohorts across the UK with respect to gender split, declared disability and first in family status [1]. Notably, the number of NU students who self-identified as belonging to one or more widening participation (WP) groups (almost 89% NU students) [27] is significantly above sector norms. Declared disability for participants of this study (Figure 2C) is 18.9%, slightly lower than institutional UG data (24%) and similar to national data (18% for UK), while first in family status is 44.1% for participants, a little higher than UK data (41%) [24]. Figure 2D shows approximately 41% respondents with no WP, indicating that approximately 59% possess one or more of the four WP characteristics investigated: socio-economic background, disability, first in family status and care experience. To ensure that cohorts with shared WP characteristics are not inadvertently disadvantaged, we need to incorporate strategies to identify barriers to cognitive and affective domain progression and be more responsive to the quickly changing landscape of education for learners. It is also vital to identify knowledge gaps and provide flexible value-based support where students can engage in self-regulated learning (SRL) early on, carry out their own needs analysis and choose appropriate supporting resources. Whilst this reflects Zimmerman’s cyclical phase model of SRL [24], the challenges of autoregulation in complex, multi-digital learning environments must also be considered and supported [47]. Therefore, implementing relevant learner-centric supporting opportunities, through strategic choice as demonstrated by the year of study-specific usage data (Figure 7), is critical in achieving this.

### Redefining engagement, confidence and student intention

Contrary to some current perceptions, there was a surprisingly high level of students feeling engaged with their course, regardless of year groups (Figure 3). This could suggest that the definition of “engagement” when used in general terms should be clarified to avoid a reductive oversimplification of how we define it, particularly focusing on the discrepancy between student and practitioner views. The concept of student engagement is broad and multifaceted, and to address engagement issues effectively, we must first clarify what we expect engagement to look like in different contexts. A recent article by WONKHE [48] considered what is meant by student engagement which prompts the questions; are we referring only to in-person involvement or could engagement be defined as simply submitting assessments on time? Attendance at synchronous sessions versus self-directed asynchronous independent working generates very different metrics. These different approaches to study and measures of engagement can in turn influence how students who do not ‘attend’ are viewed.

Again, surprisingly, overall confidence in calculations was reported by students as good, with no real differences against level of study. This higher-than-expected general confidence may be related to participant interpretation of the term ‘calculation questions’ which may be perceived as performing short calculations, rather than mathematics and analysis in general as we intended. Students expressed very high levels of intention to use the resources with 93.9% saying they were ‘very likely’ or ‘likely’ to use them (Figure 4A), with those working increasingly long hours showing slightly more intention to use the resources (Figure 4B). The most significant (*p* = 0.028) determinant of intention to use was low calculation confidence, suggesting students require more opportunity to bridge a confidence gap potentially more than a knowledge gap.

Our study found a highly significant difference between calculation confidence and the number of WP characteristics a student had (free school meals, declared disability, first in family and care experienced). The higher the number of WP characteristics, the lower the confidence: Kruskal-Wallis H = 14.507, *p* = 0.002 (exact) (Figure 3C). Therefore, WP students could benefit most from having the opportunity to engage with targeted resources to build confidence and engage more with their course, potentially improving their academic performance. In agreement with this finding, Finn et al recently published a report on differential attainment (defined as “the variation in academic achievement between groups of students with protected characteristics (including race, disability, or socioeconomic background)) and those without” [49]. They state that even when previous attainment and background factors are accounted for, disparities in students’ academic achievement persist. Importantly, Finn et al directly relate differential attainment to several themes identified through student surveys that overlap with the areas identified to impact on students in this study. These include financial insecurity; mental health and wellbeing; inaccessible physical and learning environments; curricula and assessment challenges [49].

One of the most intriguing findings of this study is the disparity between student intention and action. Despite identifying a strong intentionality to use these resources, particularly those with low confidence and those who work long hours, SW analytics show only 197 of the total 779 enrolled students (see Table 1) did use them. Of the 149 respondents to Survey B, approximately half reported that they used them (Figures 5A,B). The reasons for this discrepancy between intended use and actual use will form the basis of future work (see below).

### Improving confidence

The quantitative skills SWs have been found to play a role in building confidence, which can reduce student anxiety. This is especially valuable for those without a strong background in maths.

By revisiting foundational concepts, the SWs offer reassurance and build confidence ahead of data interpretation.

Student free text feedback (data not shown) highlights the value of clear, accessible materials in helping students feel more prepared, supporting their academic skill development and ultimately emotional wellbeing.

Level 6 students see value in the resources but struggle to engage. These students are working/caring more hours than any other year group, with 12.5% working/caring over 21 h a week (Figure 5C). Most university student workload models expect 10 h of work per credit, with 120 credits typically per year for full time students equating to 1,200 h and typically 35–40 h of study, including contact hours, per week. As a result, current credit-associated time does not allow for such a level of external commitment and will therefore undoubtedly impact students with the most financial hardship, directly affecting the student’s sense of belonging [50] as their opportunity to connect in person is lessened. The 2024 HEPI Advance HE student experience survey states that students in health subjects spend on average 55.9 h per week in combined paid work and study [51]. Students are increasingly time poor, which could account for the lower level of Level 6 students’ engagement with the resources and the surveys.

The data from Figure 5 suggest that students across the different levels of study are engaging in various strategies for choosing resources. This may contribute some insight into student learning behaviour toward learning capital gain [52]. The data from this study and previous publications [53, 54] indicate there may be a trade-off between the perceived value of attending taught sessions, versus time availability, in students deciding when and how to engage with resources. The learner’s narrative on the value of formative learning for assessment, flexible skill and confidence building, enhancing opportunities, plus general engagement challenges that we see across the sector is more important than ever. Student engagement may also be further influenced by institutional, departmental and subject specific cultural characteristics. It is crucial to understand if certain groups are more impacted and/or significantly disadvantaged by the reasons behind the shift away from traditional attendance. Could this shift further contribute to achievement and awarding gaps for already disadvantaged students?

In terms of transition to higher education, academic knowledge transfer strategies are also changing rapidly, owing partly to changes in the pipeline of educational strategy in schools and colleges, the utilisation of digital technologies and of course GenAI. It is noteworthy that whilst increased integration of GenAI holds exciting potential for efficiency of teaching and learning, there is potential to exacerbate the existing digital divide [55] caused by the financial burdens discussed in this paper, further impacting students. Students facing social and cultural challenges are taking on more external commitments that will impact their wellbeing and affective domain of learning, whereby university tasks may become low priority and even low value. These changes will undoubtedly impact the evolution of learning capital, particularly the cognitive domain of learning (e.g., process of study skills acquisition, academic knowledge gain and application). It is therefore vital for academic colleagues to be cognisant of the complex cultural, social, economic and personal challenges that combine to affect student learning.

Although a significant amount of time and effort is invested by academics to develop strategies and resources to improve engagement, knowledge acquisition and skills development, there is a discussion to be had around current practices, processes and policies. Current approaches may drive students to merely comply with what we require of them from obligation, rather than intrinsically motivated engagement. Further, academics’ view of what is deemed necessary, or essential learning practice is potentially becoming increasingly discordant with students’ value-centric triaging of their work or caring obligations. Time poor students triage their commitment to learning and assessment efficiency differently, which is particularly pertinent when studying consistently high-pressure programmes. Our data on intention versus reality demonstrate that lack of time, rather than WP characteristics, has a substantial impact on students’ availability to engage. Therefore, transparency on the relevance of tasks could be key to engagement, as discussed below.

### Barriers and value

With access and continuation data showing students from LPN behind government targets these metrics for WP students appears to be worsening [56]. Meaningful engagement is about providing value-added experiences that genuinely support learning, personal development, and long-term success. To truly understand engagement, we should acknowledge the financial pressures shaping students’ choices and their capacity to participate, as identified in our data.

It is clear from recent HEPI data [51] that students are increasingly time-poor and anxious. For many students, the cost of participating in HE is not just about tuition. Everyday essentials including food, transport and rent present significant barriers [57]. Students are increasingly pressed for time and mental space, juggling rising living costs, academic demands and part time work. More than three-quarters (78%) of students say the rising cost of living may negatively affect their academic performance, and over one-third (35%) now feel less likely to pursue further study after completing their current course [58]. Half are working 15–35 h a week and a third report struggling to balance work and study. These pressures force students to make difficult decisions about how and where they use their time [59]. Students are looking for value-added, they are not just chasing grades, they are seeking flexibility, relevance, and efficiency in how they engage. For commuting students, those with care responsibilities or demanding work schedules, online tools like LearnSci SWs are genuinely valuable because students can study effectively from home, saving both time and money. It’s not disengagement, it is a smart, strategic choice.

From our study, Level 6 students are working more hours, and the content of their final year will undoubtedly carry more weight and therefore assessments carry higher risk. The pressure of conducting more independent analysis and critical evaluation typically expected of final year students (‘they should know it by now’) for higher stakes assessments, in parallel with working more hours, means final year students need to be more, not less supported. Offering timely, optional high value supporting resources can help. Some final year students on IBMS accredited undergraduate programmes may well have secured work in connection to their clinical laboratory placement and may engage with cover or ‘bank work’. This type of shift work is notoriously inflexible and can prevent students attending timetabled sessions that clash. It seems counter intuitive that students gaining appropriate professional experience are placed at a disadvantage by missing on campus sessions. Similarly, any student who is in paid work is gaining valuable transferable professional skills, which many institutions are trying to promote and evidence because of the focus on graduate attributes. It might therefore be more appropriate to consider ways that we can recognise and reward students who are in paid employment, with work-based learning recognition, accepting content for skills portfolios or even credits.

### Engagement with Smart Worksheet resources

Students identified several reasons for not engaging more with the SWs (Table 2), pointing to both practical and communication-related barriers. A small proportion of students decided that how the resources ‘look’ whether too easy, hard or uninteresting determined any further interaction with the SWs. Without exploring the content, it would be difficult to for them to ascertain the true difficulty and so understanding what makes a positive first impression and encourages students to explore the SWs would be valuable insight. A recurring reason identified by students was lack of time with many students stating in the free text comments that they were overwhelmed with other academic demands. Other students felt they were already confident in the topic areas, choosing not to spend additional time on the SWs. This might correlate with the slight increase in confidence in calculations (Figure 3D) observed for those students who worked more hours. If students have a choice in the number of hours paid work they undertake per week, those who are more confident in their academic abilities might choose to work more hours and be able to successfully balance this against their studies. Although background information and feedback are built into each worksheet, some students said they found them confusing or needed more guidance to fully benefit. This suggests that the Supplementary Material and instructions may not have been visible or clear enough to support all users effectively. These data suggest that improved signposting to the resources and clearer instructions within the SWs could positively impact on their sustained use Students suggested strategies to improve their awareness of the resources while others asked for clarity. A simple combination of regular ‘shout-outs’ in lectures, face-to-face and recorded demonstrations, with a dedicated walkthrough of the first activity could boost visibility and familiarity. Students also suggested embedding the resources into seminars.

Promotional emails showing where to access the tools may feed into email fatigue, therefore other methods such as updates from student representatives, peer mentors or academic tutors highlighting value and importance from a student perspective could better promote such resources too. Finally, many want evidence that the resources work, asking for a comparison of marks of students who did, versus did not use the SWs. This could provide powerful motivation for other students to engage with the resources if there is a positive correlation. The authors do, however, recognise there are complex caveats with correlation and causation with this approach. Overall, raising awareness through consistent, clear communication and demonstrating value through real outcomes will be key steps toward increasing student engagement with the SWs.

In this study, raising awareness of the SWs was conducted during lectures and via email and this could potentially suggest that those who were seemingly unaware were those who do not attend lectures and or engage with email correspondence (as mentioned above). The challenge of how to reach these “absent” students remains, particularly as they likely to benefit the most from the SWs.

### Recommendations

Students with different levels of self-perceived confidence found the SWs of value noting that they had a broader impact on their learning, allowing them to identify strengths and weak points, improve confidence in calculations, encourage engagement, and feel more prepared for future assessments (Figure 6D). These data indicate that we can utilise formative yet relevant upskilling tasks to help students feel more connected and engaged with their programme. The fact that SWs were well received by all students, regardless of their confidence levels (Figures 6C,D) warrants improved strategic implementation:

- Increase Visibility and Clarity: Factor in time to discuss SWs in lectures, provide clear explanations of their purpose, and offer short demonstrations or a walkthrough of a worksheet to build familiarity.
- Integrate into Teaching: Embed SWs into relevant timetabled seminars, tutorials and/or core learning activities to help students learn how and when to use them effectively. This needs to be strategic to factor in student learning behaviour whereby most usage was most prevalent in the first week of release (Figures 6A,B).
- Show Evidence of Impact: Share data comparing academic performance and or affective domains such as confidence and satisfaction, of students who used SWs versus those who did not, to demonstrate their value and encourage engagement through proven outcomes.
- Influence agency: Providing signposting and space to support the use of pertinent resources. Utilise ‘value-mapping’ to help students correlate the resources with the skills associated with achieving intended LOs.

### Challenges

Promoting academic resources in HE is increasingly challenging, particularly with low in-person attendance at sessions. A key issue is visibility, students often forget about resources or are unsure of their purpose or benefits. In this context, traditional communication methods like emails or brief mentions in lectures may not be enough, and more active, embedded approaches are needed to truly capture attention.

When fewer students are physically present, opportunities for direct communication such as live demonstrations, reminders or walkthroughs are limited. As a result, key messages can be missed, making it harder to build awareness and highlight the value of support tools such as SWs.

Many students are already juggling heavy workloads, part-time jobs, and personal responsibilities, leaving little time to explore high value optional resources. In this context, emails or online posts often go unnoticed. To address this, promotion must move beyond traditional methods and be embedded into core learning activities and digital platforms students already engage with regularly.

### Future work

We will adopt a data-driven, evidence-based approach to further evaluate and refine the implementation of Smart Worksheets (SWs) within biomedical science education. A key objective is to develop an effective strategy for integrating these resources across other IBMS-accredited programmes, with clear guidance on how to embed structured yet flexible opportunities to engage with the SWs within academic timetables. A longitudinal study tracking student confidence, engagement, and academic performance over time would provide valuable insights into the long-term impact of SW use on learning trajectories. This will help to clarify the relationship between confidence and attainment, whilst considering cohort effects. We plan to investigate correlations between SW completion and module performance. Comparative analysis of assessment scores between users and non-users will help determine effectiveness. We will consider performance relative to other user behaviour including timing of completion relative to assessment deadlines.

Further qualitative data from surveys and focus groups -including students who did and did not engage with the SWs - may provide insight to enable a greater understanding of the first impressions that encourage student use and how to tackle other barriers.

### Limitations

Our analysis of survey B and any extrapolation is difficult, as there were a number of students who used the resources but did not complete the survey.

There may be a discrepancy between author and student interpretation of ‘calculation confidence’ which will be clarified in the next stage of the study.

A clear differentiation between confidence, anxiety and ability in analytical skill development was not established in this initial study.

## Summary table

### What is known about this subject

- Undergraduate students vary in their quantitative skill ability
- The QAA subject benchmark statement requires a commitment to inclusive practices for diverse student cohorts through considered course design.
- Flexible and personalised learning resources are required to support diverse learners in higher education.

### What this paper adds

- LearnSci Smart worksheets are a valuable resource, validated for use in Biomedical Sciences to support quantitative skill development
- This study provides insights regarding student engagement, support needs and the value of flexible learning resources in contemporary HE.

## Concluding statement

This work represents an advance in biomedical science because it addresses gaps in numerical knowledge and analytical skills, enhancing confidence in diverse student groups.

## Data Availability

The data presented in this article are not all readily available as per our ethics approval. Further enquiries should be made to s.veuger@northumbria.ac.uk.
