# Supplementary material for: Supporting quantitative skills in biomedical science with smart worksheets: intentions, impact and barriers to engagement
Source: Br J Biomed Sci. 2026 Jun 29;83:15078. doi: 10.3389/bjbs.2026.15078 (PMC13359501; doi:10.3389/bjbs.2026.15078)
Supplement: Supplementary file 1 [file DataSheet2.pdf]

Numeracy in biomedical science at Northumbria University

How can we help support your learning?

Welcome to this survey which seeks your thoughts and opinions about the the numeracy and mathematical components of your course, and how we can better support your access to, and engagement with, supporting educational resources.

All responses are anonymous and by completing the survey you are giving consent for your anonymous data to be used in the study. It should take no longer than 8-10 minutes to complete.

Who can take part?
If you have been invited to take part and are 18 years or older, you can take part.

Will my taking part be kept confidential?
All data from this survey will be anonymous and confidential. This data will be kept secure by being stored on password-protected University and LearnSci devices. If this data contributes to a publication, the data will be kept for a minimum of 5 years.

What if I don't want to answer a question?
There is no obligation to answer every question (except the initial consent question). You can simply leave the response area blank if you do not wish to answer a particular question that is being asked, and some questions additionally have a "prefer not to say" option. This includes some questions about your background, which we ask in order to understand your circumstances a little better to help us consider how best we can support all students.

What if I change my mind?
If you start the survey but then change your mind, simply do not finish the survey. You will not be able to withdraw from the survey once you have started, as responses are anonymous so it will not be possible to identify you and extract your results from everyone else's.

Who is responsible for this survey?
This survey is a co-collaboration of Stephany Veuger at Northumbria University, Sue Jones at IBMS, staff members at LearnSci (the creators of the Quantitative Skills Smart Worksheets), and selected other academic staff members.

What should I do if I have questions or concerns?
If you have any questions or concerns, contact project lead Stephany Veuger:
s.veuger@northumbria.ac.uk

* 1. **Select one of the following options:**

◯ I am 18 years old or older, and I consent to continuing with this survey.

◯ I do not consent to continuing with this survey.

Numeracy in biomedical science at Northumbria University

Numeracy in your course

2. **How confident do you feel when answering calculation questions in your course?**

◯ Very confident

◯ Fairly confident

◯ Not very confident

◯ Not at all confident

If you would like to comment further upon your answer, please specify here:

3. **Please select your level of agreement with the following statements:**

	Very strong agreement	Strong agreement	Medium agreement	Slight agreement	No agreement at all
Calculation skills are important for students studying biomedical science	◯	◯	◯	◯	◯
Calculation skills can be developed and improved with practice	◯	◯	◯	◯	◯
Needing to do calculations makes me feel anxious	◯	◯	◯	◯	◯
Applying calculations to biomedical contexts is challenging	◯	◯	◯	◯	◯
I feel engaged with the content of my course	◯	◯	◯	◯	◯

If you have any comments or further thoughts on the questions above, please write them here:

Numeracy in biomedical science at Northumbria University

Resources to support numeracy skills

4. **If an optional set of resources aimed at developing your calculation skills in biomedical contexts was available to you, do you think you would use them?**

- ◯ Very likely to use them
- ◯ Fairly likely to use them
- ◯ Fairly unlikely to use them
- ◯ Very unlikely to use them

5. **What would help encourage you to use such a resource? These could be features of the resources themselves, or how they are provided, or anything else!**

6. **What would discourage you from using them?**

Numeracy in biomedical science at Northumbria University

Finally, a little about yourself

These optional questions have been included to help us identify and meet the needs of all different types of students. Remember all answers are anonymous, stored securely and will be reported upon as a group, not individually. Your answers will not be used to identify you.

7. Which of the following best describes your education before arriving at university?

- ◯ A-levels (not including mathematics)
- ◯ A-levels (including mathematics)
- ◯ A-levels (including further mathematics)
- ◯ T-levels
- ◯ Access to HE
- ◯ International Baccalaureate (IB)
- ◯ Prefer not to say
- ◯ Other (please specify)

____

8. What is your gender?

- ◯ Male
- ◯ Female
- ◯ Non-binary or other
- ◯ Prefer not to say

9. Which best describes your student status?

Note: 'International student' means you pay International tuition fees and have moved to another country to attend university from the start of your degree.

- ◯ Home student
- ◯ International student
- ◯ Prefer not to say

10. **Do you fall under any of the categories below:**

	Yes	No	Unsure	Prefer not to say
You were eligible for financial support whilst at school (e.g. free school meals in the UK)	◯	◯	◯	◯
You have a physical or mental health disability (seen or unseen)	◯	◯	◯	◯
Your parents (or parental figures) did not go to university	◯	◯	◯	◯
You spent time in local authority care	◯	◯	◯	◯

11. **On average, how long do additional commitments (including paid work and/or caring responsibilities) currently take per week during term time?**

Your best estimate is fine, thank you. Please do not include hobbies or volunteering within this count.

- ◯ 0 hours (none)
- ◯ 1 - 5 hours
- ◯ 6 - 10 hours
- ◯ 11 - 20 hours
- ◯ 21 - 30 hours
- ◯ 31 or more hours
- ◯ Prefer not to say

If you would like to comment further upon your answer, please specify here:

You have reached the end of the survey. Thank you for your time.
