# Supplementary material for: Supporting quantitative skills in biomedical science with smart worksheets: intentions, impact and barriers to engagement
Source: Br J Biomed Sci. 2026 Jun 29;83:15078. doi: 10.3389/bjbs.2026.15078 (PMC13359501; doi:10.3389/bjbs.2026.15078)
Supplement: Supplementary file 2 [file DataSheet1.pdf]

Quantitative Skills Smart Worksheets at Northumbria - your thoughts welcome!

How well do these resources support your learning?

We would like to hear your thoughts and opinions on the **Quantitative Skills** resources (whether you have used them yet or not), and how we can better support your access to, and engagement with, supporting educational resources.

All responses are anonymous and by completing the survey you are giving consent for your anonymous data to be used in the study. It should take no longer than 8-10 minutes to complete.

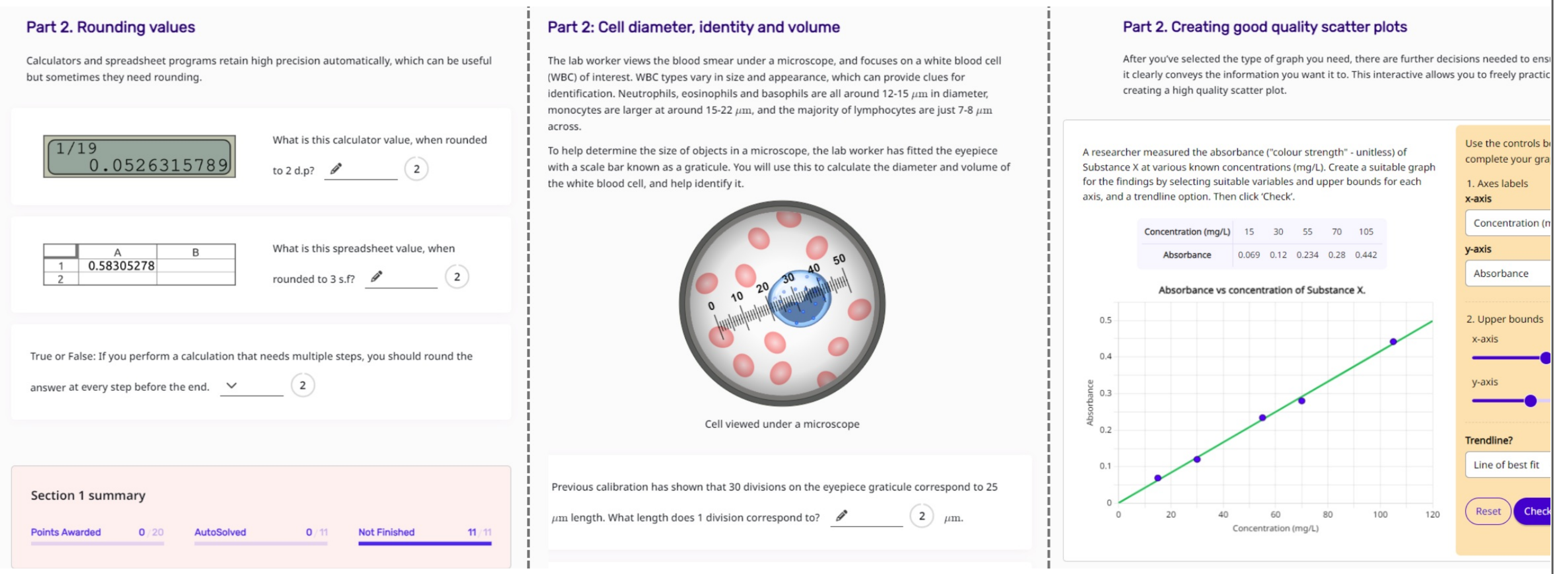

Who can take part?
If you have been invited to take part and are 18 years or older, you can take part.

Will my taking part be kept confidential?
All data from this survey will be anonymous and confidential. This data will be kept secure by being stored on password-protected University and LearnSci devices. If this data contributes to a publication, the data will be kept for a minimum of 5 years.

What if I don't want to answer a question?
There is no obligation to answer every question (except the initial consent question and one about resource usage which determines which questions you see afterwards). You can simply leave the response area blank if you do not wish to answer a particular question that is being asked, and some questions additionally have a “prefer not to say” option.

What if I change my mind?
If you start the survey but then change your mind, simply do not finish the survey. You will not be able to withdraw from the survey once you have started, as responses are anonymous so it will not be possible to identify you and extract your results from everyone else's.

Who is responsible for this survey?
This survey is a co-collaboration of Stephany Veuger at Northumbria University, Sue Jones at IBMS, staff members at LearnSci (the creators of the Quantitative Skills Smart Worksheets), and selected other academic staff members.

What should I do if I have questions or concerns?
If you have any questions or concerns, contact project lead Stephany Veuger: s.veuger@northumbria.ac.uk

* 1. **Select one of the following options:**

◯ I am 18 years old or older, and I consent to continuing with this survey.

◯ I do not consent to continuing with this survey.

Quantitative Skills Smart Worksheets at Northumbria - your thoughts welcome!

2. **There was a previous questionnaire like this, which was about your perceptions of calculation skills within your course, and how these could be supported. Did you take part in this questionnaire?**

Note you are welcome to complete this questionnaire regardless of your answer!

◯ Yes

◯ No

◯ Don't remember

Quantitative Skills Smart Worksheets at Northumbria - your thoughts welcome!

Resource usage

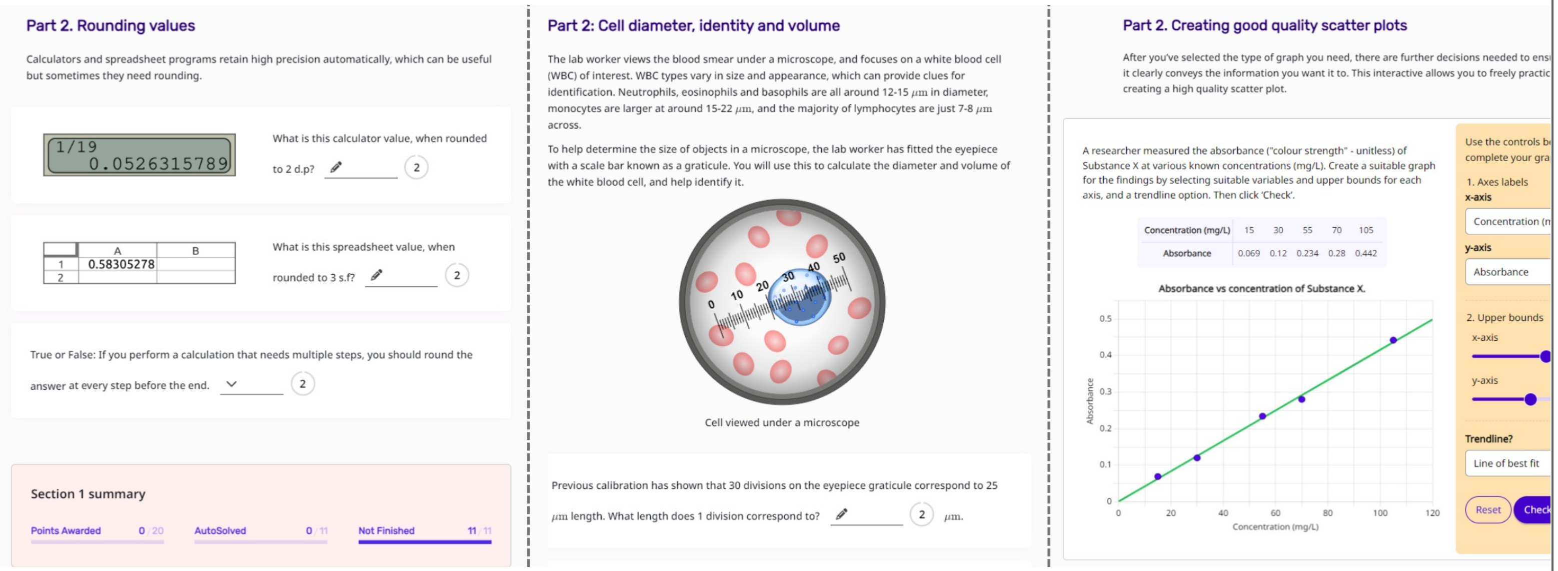

* 3. **Have you used the Quantitative Skills resources shown above?**

◯ Yes, all of them

◯ Yes, some of them

◯ No (or not yet)

Quantitative Skills Smart Worksheets at Northumbria - your thoughts welcome!

Which resources?

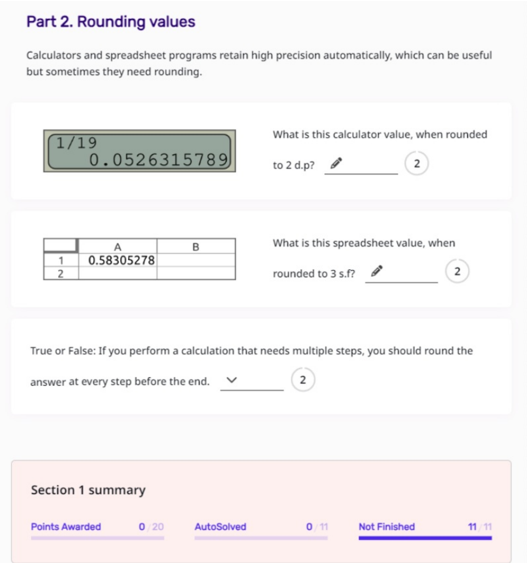

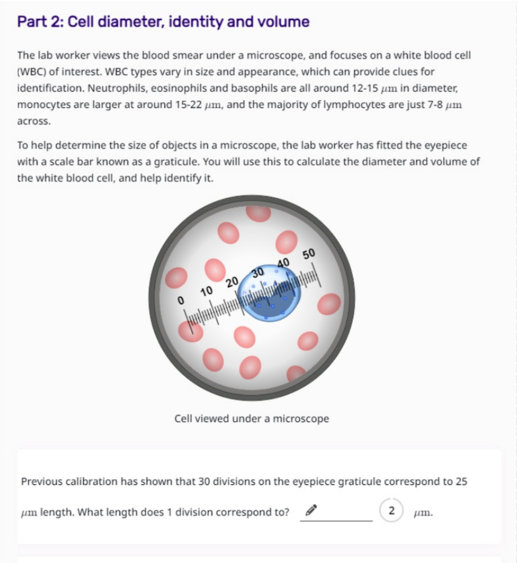

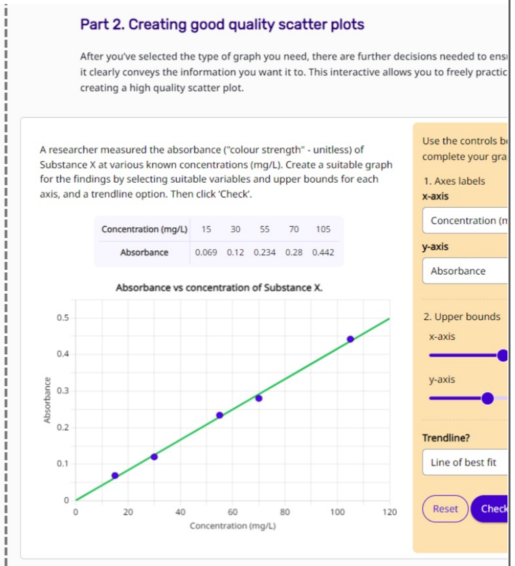

4. **As best as you can remember, which of these Quantitative Skills resource did you use, either partially or fully?**

	I finished this one	I got partway through this one	I didn't start this one	I don't remember
Quant Skills 1: Displaying numbers scientifically	◯	◯	◯	◯
Quant Skills 2: Units and unit conversions	◯	◯	◯	◯
Quant Skills 3: Scientific formulae	◯	◯	◯	◯
Quant Skills 4: Data visualisation and graphing	◯	◯	◯	◯
Quant Skills 5: Averages, spread and precision	◯	◯	◯	◯

5. **We would value further information about your answers above, why you used or didn't use resources from this set.**

Quantitative Skills Smart Worksheets at Northumbria - your thoughts welcome!

Your thoughts welcome! Page 1 of 3

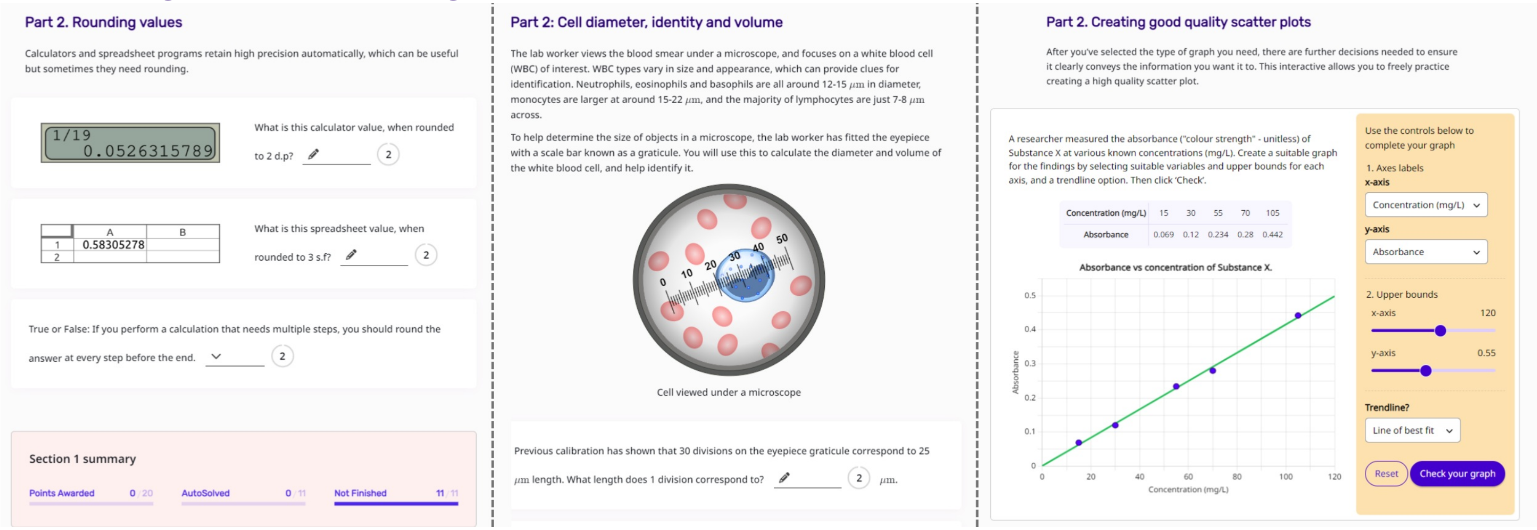

6. **Before starting the Quantitative Skills resources, how confident did you feel when answering calculation questions in your course?**

- ◯ Not at all confident
- ◯ Not very confident
- ◯ Fairly confident
- ◯ Very confident

If you would like to comment further upon your answer, please specify here:

[ ]

7. **What do you think of the overall challenge level of the Quantitative Skills resources?**

- ◯ Much too difficult
- ◯ A little too difficult
- ◯ About right for me
- ◯ A little too easy
- ◯ Much too easy

Other / a mix (please specify below)

8. **Regarding their usefulness, how much do you agree that the Quantitative Skills resources:**

	Strongly agree	Agree	Neither agree nor disagree	Disagree	Strongly disagree
...are straightforward to use and interact with	◯	◯	◯	◯	◯
...used topics and situations which felt relevant to my degree	◯	◯	◯	◯	◯
...provided helpful feedback for correcting mistakes and misunderstandings	◯	◯	◯	◯	◯

If you have any comments or further thoughts on the questions above, please write them here:

9. **Regarding their broader impact, how much do you agree that the Quantitative Skills resources:**

	Strongly agree	Agree	Neither agree nor disagree	Disagree	Strongly disagree
...helped me identify strengths and weak points in my learning	○	○	○	○	○
...made me feel more confident in my calculation abilities	○	○	○	○	○
...helped me engage more with the module overall	○	○	○	○	○
...helped me feel more prepared for future assessments	○	○	○	○	○

If you have any comments or further thoughts on the questions above, please write them here:

Quantitative Skills Smart Worksheets at Northumbria - your thoughts welcome!

10. **How soon after first hearing about the Quantitative Skills resources did you start using them?**

- ◯ Within 1 day
- ◯ Within 1 week
- ◯ Within 2 weeks
- ◯ Within 1 month
- ◯ Longer than a month

If you would like to share more about when you chose to start the resources when you did, please let us know here:

11. **Have you, or will you, take any of the Quantitative Skill resources multiple times for additional practice or revision?**

- ◯ **I have repeated** at least one of the resources and **intend** to repeat more
- ◯ **I have repeated** at least one of the resources but **do not intend** to repeat more
- ◯ **I have not repeated** any of the resources but **intend** to repeat some in the future
- ◯ **I have not repeated** any of the resources and **do not intend** to repeat some in the future

If you have further thoughts on this topic, please let us know here:

12. **Were there any topic or skill areas you believed were missing from the Quantitative Skills resources? If so, what?**

Quantitative Skills Smart Worksheets at Northumbria - your thoughts welcome!

Your thoughts welcome! Page 3 of 3

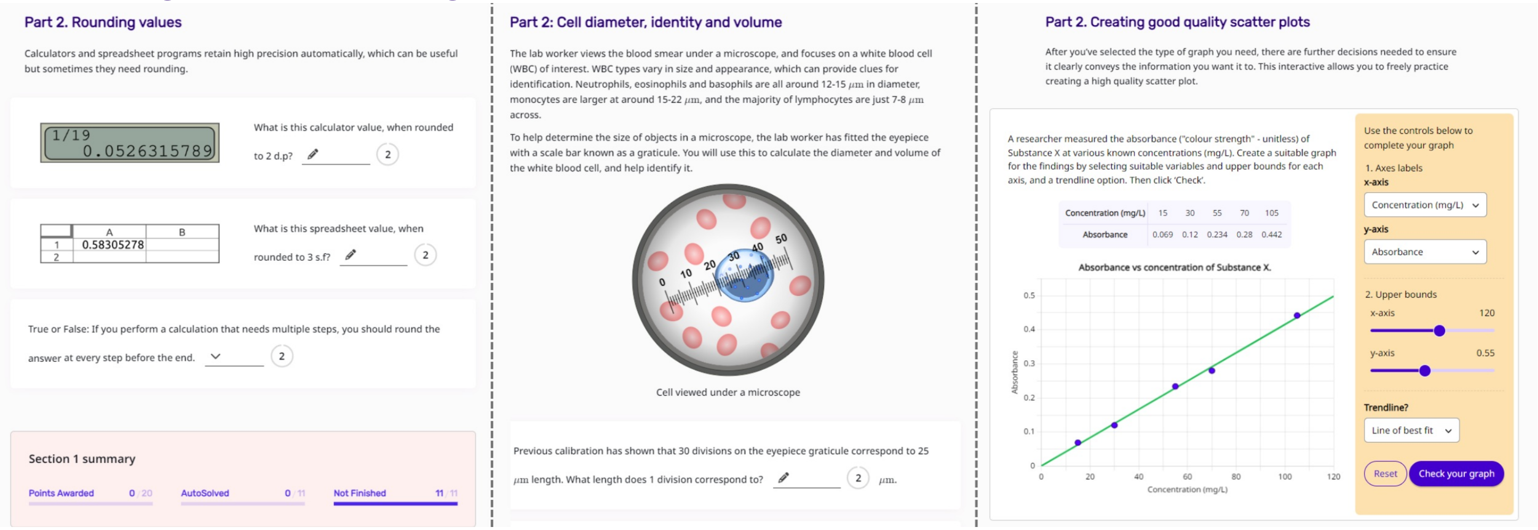

13. **On a scale of 1 to 10, how much would you recommend the Quantitative Skills resources to other students on a similar course?**

1	2	3	4	5	6	7	8	9	10
☆	☆	☆	☆	☆	☆	☆	☆	☆	☆

14. **Please explain why you gave the Quantitative Skills resources this score.**

Quantitative Skills Smart Worksheets at Northumbria - your thoughts welcome!

Please tell us more!

15. **Please tell us why you didn't use the Quantitative Skills resources, or haven't used them yet. Check all that apply.**

- [ ] I didn't have time
- [ ] They looked too difficult
- [ ] They looked too easy
- [ ] They didn't look interesting
- [ ] I couldn't open or use them
- [ ] I didn't know about them
- [ ] I couldn't find them
- [ ] Other (please tell us why)

16. **How likely are you to use the Quantitative Skills resources in the future?**

- ◯ Very likely
- ◯ Likely
- ◯ Unlikely
- ◯ Very unlikely
- ◯ Don't know

17. **Do you have any suggestions for what might increase the likelihood of students using the Quantitative Skills resources?**

Quantitative Skills Smart Worksheets at Northumbria - your thoughts welcome!

Finally, a little about yourself

These optional questions have been included to help us identify and meet the needs of all different types of students. Remember all answers are anonymous, stored securely and will be reported upon as a group, not individually. Your answers will not be used to identify you.

18. **Which of the following best describes your education before arriving at university?**

- ◯ A-levels (not including mathematics)
- ◯ A-levels (including mathematics)
- ◯ A-levels (including further mathematics)
- ◯ Access to HE
- ◯ International Baccalaureate (IB)
- ◯ Prefer not to say
- ◯ Other (please specify)

19. **What is your gender?**

- ◯ Male
- ◯ Female
- ◯ Non-binary or other
- ◯ Prefer not to say

20. **Which best describes your student status?**

Note: 'International student' means you pay International tuition fees and have moved to another country to attend university from the start of your degree.

- ◯ Home student
- ◯ International student
- ◯ Prefer not to say

21. **Do you fall under any of the categories below:**

	Yes	No	Unsure	Prefer not to say
You were eligible for financial support whilst at school (e.g. free school meals in the UK)	◯	◯	◯	◯
You have a physical or mental health disability (seen or unseen)	◯	◯	◯	◯
Your parents (or parental figures) did not go to university	◯	◯	◯	◯
You spent time in local authority care	◯	◯	◯	◯

22. **On average, how long do additional commitments (including paid work and/or caring responsibilities) currently take per week during term time?**

Your best estimate is fine, thank you. Please do not include hobbies or volunteering within this count.

- ◯ 0 hours (none)
- ◯ 1 - 5 hours
- ◯ 6 - 10 hours
- ◯ 11 - 20 hours
- ◯ 21 - 30 hours
- ◯ 31 or more hours
- ◯ Prefer not to say

If you would like to comment further upon your answer, please specify here:

You have reached the end of the survey. Thank you for your time.
